# Supplementary material for: Physiological responses of Cucurbita pepo seeds to cadmium and copper stress: Differential impacts on reserve mobilization, metabolic efficiency, and growth
Source: PLoS One. 2026 Jan 30;21(1):e0341814. doi: 10.1371/journal.pone.0341814 (PMC12858001; doi:10.1371/journal.pone.0341814)
Supplement: Attachment — Submitted filename: Rebuttal letter_Acila et al.pdf [file pone.0341814.s003.pdf]

October 1, 2025

Rebuttal Letter

Dear Professor Debasis Mitra,

Ref: PONE-D-25-40701

Thank you for giving us the opportunity to revise our manuscript titled **"Physiological responses of *Cucurbita pepo* seeds to cadmium and copper stress: Differential impacts on reserve mobilization, metabolic efficiency, and growth."**

We are grateful to you and the reviewers, **Dr. Aishwarya Singh** and **Dr. Ravikumar D. Dodiya** for the constructive comments and valuable suggestions, which have significantly helped us improve the quality and clarity of our manuscript.

We have carefully addressed all the points raised during the review process. The main revisions to the manuscript are summarized below:

1. Responses to Reviewer #1's Comments:

- **Clarification of the research gap:** As suggested, we have explicitly stated the specific knowledge gap and the significance of our study in the **Introduction** section (newly added paragraph).
- **Critical analysis of limitations:** A new subsection titled **"Study Limitations and Future Directions"** has been added to the **Discussion** to critically address the study's limitations, such as the controlled laboratory conditions and the focus on early growth stages.
- **Citation and Reference Style:** The reference style has been thoroughly updated to conform to PLOS ONE's Vancouver style (numbered, in square brackets). The reference list has been checked for completeness and accuracy.
- **Language and Structure:** The entire manuscript has undergone comprehensive language editing to correct grammatical errors and improve sentence structure, coherence, and flow. We confirm that the manuscript is now written in standard English.

2. Responses to Reviewer #2's Comments:

- **Abstract:**
    - Long sentences have been split for better clarity.
    - The presentation of results has been made more parallel, primarily using the "% increase/decrease" format.
    - The phrasing regarding PCA has been simplified as recommended.

- The IMRaD structure has been sharpened, and the suggested keywords ("Heavy metal stress," "Principal component analysis") have been added.

- **Materials and Methods:**
  - Redundant sentences (e.g., regarding growth measurements) have been merged and simplified.
  - The notation "µM" is now used consistently throughout the text.
  - A space has been consistently added between numbers and units (e.g., "10 ml").
  - All cited methods are now fully referenced in the bibliography.
  - Subsection titles have been clarified (e.g., "Protein quantification in cotyledons").
  - The statistical analysis description has been rewritten for precision and now mentions that data were tested for significance using Fisher's LSD test at $p < 0.05$.

3. Compliance with Journal Requirements:

- **Funding Statement:** As requested, all funding-related text has been removed from the **Acknowledgements** section. We would like to clarify that the absence of specific funding was explicitly mentioned in the original manuscript to ensure full transparency. The funding statement in the submission system remains accurate and unchanged as: **"The author(s) received no specific funding for this work."**
- **Style Requirements:** The manuscript has been formatted to meet PLOS ONE's style requirements, including the correct file naming format for the revised submission.
- **Data Availability:** We confirm that all data underlying the findings are fully available within the manuscript and its supporting information files.

We believe that our revisions have thoroughly addressed all the concerns raised, and the manuscript is now significantly strengthened. We are hopeful that the revised version meets the high publication standards of *PLOS ONE*.

Thank you for your time and consideration.

Sincerely,

Dr. Smail Acila
On behalf of all co-authors
Corresponding Author
Email: smailacila@gmail.com / smail-acila@univ-eloued.dz
