# Supplementary material for: Physiological responses of Cucurbita pepo seeds to cadmium and copper stress: Differential impacts on reserve mobilization, metabolic efficiency, and growth
Source: PLoS One. 2026 Jan 30;21(1):e0341814. doi: 10.1371/journal.pone.0341814 (PMC12858001; doi:10.1371/journal.pone.0341814)
Supplement: Attachment — Submitted filename: Response_to_Reviewers_R2.pdf [file pone.0341814.s004.pdf]

December 21, 2025

Rebuttal letter

Subject: Response to Reviewers - Manuscript ID: PONE-D-25-40701R1

To: The Academic Editor and Reviewers of PLOS ONE

Dear Dr. Debasis Mitra and esteemed Reviewers,

Thank you for considering our manuscript, **"Physiological responses of *Cucurbita pepo* seeds to cadmium and copper stress: Differential impacts on reserve mobilization, metabolic efficiency, and growth"** (ID: PONE-D-25-40701R1), and for your constructive feedback. We sincerely appreciate the time and effort you have dedicated to reviewing our work. The comments were insightful and have significantly helped us improve the manuscript's clarity, methodological rigor, and scientific depth.

We have carefully addressed all points raised by the reviewers (particularly Reviewer 1, whose detailed comments guided major revisions) and the academic editor. A point-by-point response is provided below, with all corresponding changes highlighted in the tracked-changes version of the manuscript.

Response to Reviewer 1

Comment 1 (Major): Unclear experimental unit and replication — risk of pseudoreplication.

Response: We sincerely thank the reviewer for this critical methodological observation. We agree that clarity on the experimental unit is essential for statistical validity. We have now explicitly defined the experimental unit and the analytical approach throughout the **Materials and Methods** (Section 1.4) and **Results**.

- **Clarification Added:** We have stated: "All experiments were conducted with three independent biological replicates (n = 3), where each replicate consisted of a Petri dish containing 50 seeds. For growth parameters (lengths and dry weights), 20 seedlings were randomly selected from each replicate, and their values were averaged to obtain a single mean value per biological replicate prior to statistical analysis. This approach ensured that the Petri dish was maintained as the experimental unit, thus avoiding pseudoreplication."
- **Analysis Clarified:** All statistical tests (ANOVA, LSD) were performed using the three mean values (one per biological replicate/Petri dish) for each treatment. We have updated the figure legends and table footnotes to consistently indicate "n=3 independent experiments."

Comment 2 (Major): Methodological details incomplete/ambiguous.

Response: We have expanded the **Materials and Methods** section to provide the requested details.

- **Seed Disinfection:** Clarified: "disinfected in 10% (v/v) sodium hypochlorite solution (prepared from commercial NaClO containing 12° chlorometric degree) for 10 minutes."
- **Cold Soak Rationale:** Explained its purpose: *"to synchronize germination, following our previously validated method [15]."*
- **Treatment Application:** Clarified: *"The solutions were completely replaced daily to maintain consistent metal concentrations throughout the 8-day germination period."*
- **Sample Drying for Biochemistry:** To preserve metabolite integrity, we have changed the protocol: *"All samples were freeze-dried (lyophilization) to preserve metabolic integrity..."* (Sections 1.3.4 and 3.4).
- **Sugar Extraction Details:** Provided justification and refined the protocol: *"The methanol extraction method was selected for its efficiency in extracting soluble sugars while minimizing interference from other compounds [24, 25]."* We also specified storage at 4°C and evaporation under nitrogen.

Comment 3 (Major): Missing direct measurements of metal uptake / internal concentrations.

Response: We acknowledge this as a valuable suggestion for future research. In the revised **Discussion** (Section 3.9, "Study limitations and future directions"), we explicitly state: *"First, the internal concentrations of Cd and Cu in different seed tissues were not directly measured. Future studies incorporating ICP-MS analysis would provide definitive evidence regarding metal uptake and distribution patterns."*

Furthermore, we have tempered mechanistic claims throughout the text, replacing definitive statements (e.g., "demonstrates," "causes") with more cautious language (e.g., "suggests," "may indicate," "could be attributed to") to accurately reflect the inferential nature of our conclusions regarding internal metal action.

Comment 4 (Major): Claims extend beyond the data.

Response: We thank the reviewer for this important note. We have thoroughly revised the **Discussion** to ensure our interpretations remain closely tied to our measured parameters (germination, growth, reserve mobilization, sugars, proteins, PCA).

- We have softened language regarding specific enzymatic mechanisms (e.g., α-amylase, protease inhibition) where we did not perform direct assays. We now present these as plausible interpretations supported by literature, clearly stating in Section 3.6: *"While the current study did not directly measure hydrolytic enzyme activities... the metabolic patterns observed align with established mechanisms..."*

- We have integrated references to our **companion study [15]**, which did measure oxidative stress and antioxidant enzyme activities on the same plant material under identical conditions, providing a stronger, data-backed foundation for discussing broader stress responses.

Minor Concerns:

- **Concentration Relevance:** We have added context in the legend for **Fig 1**, citing literature **[17, 18]** showing that heavy metal concentrations in contaminated agricultural soils can reach levels relevant to our chosen experimental range.
- **Calibration Curves:** We have added the linear regression coefficients (r^2 values) for both the sugar and protein standard curves in Section 1.3.4.
- **Language and Proofreading:** The manuscript has been meticulously proofread by the authors and a professional English language editing service to correct grammatical errors, eliminate repetition, and improve overall clarity and flow.

Response to Reviewer 2

We thank Reviewer 2 for their initial assessment that all comments from the previous round were addressed and for their positive feedback on the technical soundness, statistics, data availability, and presentation of the manuscript.

We believe that the comprehensive revisions made in response to Reviewer 1 have further strengthened the manuscript, enhancing its methodological transparency and interpretive rigor to meet the high standards of PLOS ONE.

We are confident that the revised manuscript is now significantly improved and fully addresses all the concerns raised. We hope you find it suitable for publication in **PLOS ONE**.

Thank you again for your guidance.

Sincerely,

Smail ACILA (on behalf of all co-authors)
Corresponding Author
Email: smailacila@gmail.com
