# Supplementary material for: Physiological responses of Cucurbita pepo seeds to cadmium and copper stress: Differential impacts on reserve mobilization, metabolic efficiency, and growth
Source: PLoS One. 2026 Jan 30;21(1):e0341814. doi: 10.1371/journal.pone.0341814 (PMC12858001; doi:10.1371/journal.pone.0341814)
Supplement: Attachment — Submitted filename: Suggestion for Plos One.docx [file pone.0341814.s001.docx]

**Physiological responses of Cucurbita pepo seeds to cadmium and copper stress: Differential impacts on reserve mobilization, metabolic efficiency, and growth**

**Suggestions for Improvement**

**Abstract**

- Some sentences are long and packed with multiple results, which may overwhelm the reader. Splitting into shorter sentences will improve clarity.
- You sometimes present results as % reduction (SVIW, seedling length) and sometimes as absolute or relative increases (proteins, sugars). Consider keeping the format parallel (e.g., always “increased by X%” / “decreased by Y%”).
- The phrase *“Cu at 100 µM transiently elevated cotyledon sugars (+63%), reflecting its dual role as a micronutrient and stressor”* is good, but a bit dense. Simplify: *“At 100 µM, Cu increased cotyledon sugars by 63%, suggesting its dual role...”*.
- The multivariate analysis (PCA) could be explained more directly. Instead of *“PCA: 79.7% variance, F1”*, say *“Principal component analysis showed Cd explained 79.7% of the variance in metabolic disruption”*.
- **Abstract structure**: *PLOS ONE* prefers IMRaD (Introduction, Methods, Results, and Discussion/Conclusion) style even in abstracts. This is mostly there, but can be sharpened.
- **Keywords**: You might add **“Principal component analysis”** or **“Heavy metal stress”** for indexing purposes.

**Material and Method section**

- Some sentences are very long and repetitive (e.g., “Radicle, hypocotyl and total seedling length: measured…” appears twice). Merge or simplify.
- Avoid redundancy—methods should be precise but concise.
- Write µM consistently (avoid mix of μM and µM).
- Always keep a space between number and unit (e.g., “10 ml”, not “10ml”).
- Ensure all cited methods (e.g., Pereira et al. 2015; Rao and Sinha 1993; Andrade et al. 2019) are fully referenced in the bibliography.
- The methods are already divided into subsections, which is good. But some subsection titles can be made clearer, e.g., instead of *“Protein content of cotyledons”*, write *“Protein quantification in cotyledons”*.
- Instead of *“to present means ± SE and perform LSD test”*, write: *“Data were expressed as means ± SE, and differences among treatments were tested using LSD at p < 0.05.”*
- Mention assumptions if checked (normality, homogeneity of variance).
- Top of Form
- Bottom of Form
